# Does Particulate Air Pollution Contribute to Infant Death? A Systematic Review

**DOI:** 10.1289/ehp.6857

**Published:** 2004-06-03

**Authors:** Svetlana V. Glinianaia, Judith Rankin, Ruth Bell, Tanja Pless-Mulloli, Denise Howel

**Affiliations:** Public Health Research Group, School of Population and Health Sciences, Faculty of Medical Sciences, University of Newcastle, Newcastle upon Tyne, United Kingdom

**Keywords:** infant mortality, particulate air pollution, postneonatal respiratory mortality, sudden infant death syndrome, systematic review

## Abstract

There is now substantial evidence that both short- and long-term increases in ambient air pollution are associated with increased mortality and morbidity in adults and children. Children’s health is particularly vulnerable to environmental pollution, and infant mortality is still a major contributor to childhood mortality. In this systematic review we summarize and evaluate the current level of epidemiologic evidence of an association between particulate air pollution and infant mortality. We identified relevant publications using database searches with a comprehensive list of search terms and other established search methods. We included articles in the review according to specified inclusion criteria. Fifteen studies met our inclusion criteria. Evidence of an association between particulate air pollution and infant mortality in general was inconsistent, being reported from locations with largely comparable pollution levels. There was some evidence that the strength of association with particulate matter differed by subgroups of infant mortality. It was more consistent for post-neonatal mortality due to respiratory causes and sudden infant death syndrome. Differential findings for various mortality subgroups within studies suggest a stronger association of particulate air pollution with some causes of infant death. Research is needed to confirm and clarify these links, using the most appropriate methodologies for exposure assessment and control of confounders.

The historic 1952 pollution episode in London, when a rapid increase in smog led to dramatic increases in daily mortality, including infant mortality (Her Majesty’s Public Health Service 1954), stimulated early studies into the effect of air pollution on population health. There is now widespread evidence that short-term increases in ambient air pollution increase mortality and morbidity in adults and children, even at exposure levels below the World Health Organization (WHO) Air Quality Guidelines for Europe, and U.S. Environmental Protection Agency (EPA) standards (Brunekreef et al. 1995; Committee on the Medical Effects of Air Pollution 1998; Dockery and Pope 1994; Holgate et al. 1999; Katsouyanni et al. 1997; Künzli et al. 2000; Lebowitz 1996; Pope et al. 1995; Samet et al. 2000; Schwartz 1994a; Sydbom et al. 2001; U.S. EPA 1987; WHO 1987). The findings are particularly consistent for particulate air pollution; most of the current evidence is available for inhalable particulate air pollution [particulate matter (PM) with a 50% cutoff aerodynamic diameter < 10 μm (PM_10_) and < 2.5 μm (PM_2.5_)] (Dominici et al. 2003; Katsouyanni et al. 1997). The effects were found to be stronger in susceptible population groups, such as the elderly, young children, and people with preexisting cardiovascular and respiratory conditions (Gouveia and Fletcher 2000; Pope 2000; Saldiva et al. 1995; Schwartz 1994b). Long-term exposure to particulate air pollution has also been associated with increases in total mortality and in cardio-pulmonary mortality and respiratory morbidity (European Environment Agency 2003; Pope 2000; Pope et al. 1995). The large overall impact of air pollution on human health and a nonthreshold linear relationship with some health outcomes (e.g., mortality and hospital admission) have prompted the WHO to put air pollution and its health effects on its agenda. It also led the U.S. EPA to draft its 2003 criteria document, which forms the basis for reevaluating the current U.S. ambient air quality standards for PM (U.S. EPA 2003; WHO 2002).

The health of infants and children is particularly vulnerable to environmental pollution and is the focus of the recently published European Environment and Health Strategy (European Comission 2003). Infant mortality remains the major contributor to childhood mortality worldwide, despite significant declines over the last two decades. Infant mortality rates vary considerably across regions and population groups, and the reasons for this variation are not fully understood. Environmental exposures, including ambient air pollution, may account partly for excesses in infant deaths. We undertook a systematic review to summarize the epidemiologic evidence for an association between levels of particulate air pollution and infant outcomes. This work was part of a broader systematic review of the association between ambient air pollution and fetal (Glinianaia et al. 2004) and infant health outcomes.

## Materials and Methods

### Identification of publications and review process.

Our methods were based on the guidelines published by the U.K. National Health Service Centre for Reviews and Dissemination (2001). We identified relevant publications using database searches with a comprehensive list of search terms and other established search methods (for details, see Glinianaia et al. 2004).

The inclusion criteria for articles were *a*) nonaccidental exposure to directly measured PM; *b*) an infant (during the first year of life) health outcome; *c*) publication between 1 January 1966 and 31 December 2003 in the English language; and *d*) availability through the British Library (London, UK) or the Internet. Articles describing outcomes related to occupational or accidental exposure were excluded, as were articles that were available as abstracts only. Only one relevant article on infant morbidity was identified by our comprehensive literature search (Gehring et al. 2002); all others explored infant mortality as an infant health outcome.

Those articles meeting the inclusion criteria were appraised by pairs of reviewers using a piloted data extraction form based on previous reviews (Bell et al. 1998; Rankin et al. 1998). We extracted information on study design, measurement methods for pollutants and outcomes, statistical techniques, confounding factors, and results.

### Exposure measurements.

Most reviewed studies used total suspended particulates (TSP) (Bobak and Leon 1992, 999b; Chay and Greenstone 1999, 2003; Ha et al. 2003; Hunt and Cross 1975; Joyce et al. 1989; Lave and Seskin 1972; Penna and Duchiade 1991; Shinkura et al. 1999), PM_10_ (Lipfert et al. 2000; Woodruff et al. 1997), or PM_2.5_ (Gehring et al. 2002; Loomis et al. 1999) as measures of exposure to PM. One study used visibility as a measure of particulate air pollution (Knöbel et al. 1995). Where possible, we recalculated effect estimates (odds and risk ratios, mean changes) as the expected change in outcome for an increase in air pollution levels by 10 μg/m^3^ (TSP, PM_10_, PM_2.5_). This facilitated comparison across studies using the same particle size measurements.

### Infant mortality.

The following definitions were used as reported in the studies: Infant mortality is the number of deaths within the first year of life per 1,000 live births; neonatal mortality (NM), the number of neonatal deaths within 0–27 days of life per 1,000 live births; postneonatal mortality (PNM), the number of deaths between 28 days and 1 year of life per 1,000 live births (Bobak and Leon 1992, 1999b; Lipfert et al. 2000) or per 1,000 neonatal survivors (Woodruff et al. 1997). Infant deaths are conventionally divided into neonatal and postneonatal deaths. The underlying causes of death differ in these time periods; in particular, preterm birth is the largest contributor to neonatal death (Maternal and Child Health Research Consortium 2001). Many reviewed studies also categorized mortality by cause of death (definitions are given in Table 1).

### Study design.

A study was described as ecologic if both outcome and exposure data were measured at a geographic area–based level. A study was described as time series when an ecologic study was based in one geographically defined population with data collected at different points in time. In semi-individual studies (cohort, case–control, and cross-sectional), outcome data were collected at an individual level and air pollution data were measured at an area-based level.

Although considered, a meta-analysis was not undertaken due to the heterogeneity of methodologies used in the studies. The results are summarized narratively with 95% confidence intervals (CIs) for estimates where possible. Given the different ways in which the studies have reported results, we could consistently report only whether any association or difference was statistically significant at the 5% level.

## Results

### Study methods.

Fifteen studies met our inclusion criteria, and the findings of the 14 articles addressing mortality are summarized in Table 1. Key aspects of study quality (i.e., study design, sample size, control for confounders) are also reported. Table 1 gives estimates unadjusted for other pollutants because there was no consistency as to whether associations with PM were reported after adjustment for other pollutants.

The studies varied by design, geographic setting, PM source and composition, copollutant exposures, exposure period investigated, and outcome. Ten studies were ecologic or time series (Bobak and Leon 1992; Chay and Greenstsone 1999 Chay and Greenstsone 2003; Ha et al. 2003; Joyce et al. 1989; Knöbel et al. 1995; Lave and Seskin 1972; Loomis et al. 1999; Penna and Duchiade 1991; Sinkura et al. 1999), two were cross-sectional (Hunt and Cross 1975; Lipfert et al. 2000), two were cohort studies (Gehring et al. 2002; Woodruff et al. 1997), and one a matched case–control study (Bobak and Leon 1999b). All used area-based estimates of air pollution exposure, except for the German study, which used a Geographic Information Systems model to provide individual ambient pollution estimates (Gehring et al. 2002). Thirteen studies used direct measurements of PM from routine monitoring of the ambient air pollution level by monitoring stations in the study areas (Bobak and Leon 1992, 1999b; Chay and Greenstone 1999, 2003; Ha et al. 2003; Hunt and Cross 1975; Joyce et al. 1989; Lave and Seskin 1972; Lipfert et al. 2000; Loomis et al. 1999; Penna and Duchiade 1991; Sinkura et al. 1999; Woodruff et al. 1997), and one study used visibility as a measure of particulate air pollution, which was reported to be highly correlated with PM_10_ levels (Knöbel et al. 1995).

Five of the ecologic and time-series studies used the annual mean concentrations of particles (Bobak and Leon 1992; Chay and Greenstone 1999, 2003; Lave and Seskin 1972; Penna and Duchiade 1991), whereas the others used means over other periods (Joyce et al. 1989; Shinkura et al. 1999) or investigated different periods before death (Ha et al. 2003; Knöbel et al. 1995; Loomis et al. 1999). In the case–control study, exposure was assigned as the mean of all 24-hr particulate air pollution measurements for the period between birth and death of the index case (Bobak and Leon 1999b), whereas in the U.S. cohort study, the mean of the PM levels for the first 2 months of life was used (Woodruff et al. 1997). In the two cross-sectional studies, the exposure period was not specified in one (Hunt and Cross 1975), whereas the other used the annual mean of PM_10_ (Lipfert et al. 2000). In the German cohort study on respiratory morbidity, the estimated annual averages of PM_2.5_ were used (Gehring et al. 2002).

Adjustments for some maternal and socioeconomic factors were made by a number of studies (Table 1); a few also adjusted for maternal smoking (Gehring et al. 2002; Lipfert et al. 2000; Woodruff et al. 1997), other air pollutants (Bobak and Leon 1992, 1999b; Lipfert et al. 2000; Loomis et al. 1999), and/or season/ weather (Chay and Greenstone 1999; Ha et al. 2003; Knöbel et al. 1995; Lave and Seskin 1972). One older cross-sectional study did not adjust for any confounding factors (Hunt and Cross 1975). Considering the comparative precision of the exposure measurements and the adjustment for key confounding factors in mortality studies, the Czech case–control study (Bobak and Leon 1999b) and the U.S. cohort study (Woodruff et al. 1997) used the strongest designs, and their results are highlighted in the findings below.

### Study findings.

#### Mortality outcomes: infant mortality.

The eight studies exploring PM and total infant mortality found little evidence of a consistent association (Table 1). Five studies of varying designs reported some positive associations (Chay and Greenstone 2003; Hunt and Cross 1975; Lave and Seskin 1972; Lipfert et al. 2000; Loomis et al. 1999), although the strength of evidence and critical exposure period differed. Three other studies (Bobak and Leon 1999b; Chay and Greenstone 1999; Penna and Duchiade 1991) reported non-significant associations. The case–control study (Bobak and Leon 1999b) found little evidence of any association with TSP levels [odds ratio (OR) = 1.03; 95% CI, 0.99–1.06].

The three studies investigating infant mortality due to respiratory causes reported a significant association with PM (Bobak and Leon 1999b; Lipfert et al. 2000; Penna and Duchiade 1991) but used different measures of effects. The case–control study (Bobak and Leon 1999b) reported a weak association with TSP levels (OR = 1.12; 95% CI, 1.01–1.28). These three studies also reported total infant mortality, and the strength of association was consistently lower than for respiratory mortality, although no formal comparisons were made.

The single study reporting infant mortality due to nonrespiratory causes found no significant association between PM levels and mortality due to this cause (Bobak and Leon 1999b) (OR = 1.01; 95% CI, 0.98–1.05), in contrast to their more positive findings for respiratory mortality.

#### Neonatal mortality.

Total NM did not show a consistent association with PM, with one U.S. study reporting a positive association (Lipfert et al. 2000), two studies with borderline findings (Bobak and Leon 1992; Chay and Greenstone 1999), and five studies from different geographic settings reporting no evidence of an association (Bobak and Leon 1999b; Chay and Greenstone 2003; Joyce et al. 1989; Lave and Seskin 1972; Shinkura et al. 1999).

The case–control study was the only one to explore NM due to both respiratory and non-respiratory causes. It reported little evidence of an association between TSP levels and either type of NM: respiratory, OR = 0.93 (95% CI, 0.67–1.32); nonrespiratory, OR = 1.00 (95% CI, 0.96–1.06) (Bobak and Leon 1999b). Another study examining respiratory NM reported a significant association with PM_10_ similar in strength to the association reported for total NM (Lipfert et al. 2000).

#### Postneonatal mortality.

Four of five studies investigating a relationship between PM and total PNM, including a cohort study (OR = 1.04; 95% CI, 1.02–1.07) (Woodruff et al. 1997), reported significant positive associations (Bobak and Leon 1992; Ha et al. 2003; Lipfert et al. 2000). The case–control study did not find a significant association (OR = 1.04; 95% CI, 0.99–1.10) (Bobak and Leon 1999b); the difference between this and the cohort study was in the precision of the estimates. Two (Bobak and Leon 1999b; Lipfert et al. 2000) of the five studies explored PNM in addition to total infant and total NM and reported similar strengths of association for all these mortality types.

In all studies examining both total and respiratory PNM (Bobak and Leon 1992, 1999b; Ha et al. 2003; Lipfert et al. 2000; Woodruff et al. 1997), the association between PM level and respiratory mortality was statistically significant and stronger than for total mortality, although no formal comparisons were made. This was true for infants of normal birth weight in the cohort study, but the results were inconclusive for the subgroup of infants with low birth weight (Woodruff et al. 1997). In the two studies where both postneonatal respiratory and nonrespiratory mortalities were investigated, there was little evidence of an association between PM levels and nonrespiratory mortality (Bobak and Leon 1999b; Woodruff et al. 1997).

Sudden infant death syndrome (SIDS) was found to be associated with ambient PM concentrations in two U.S. studies (Lipfert et al. 2000; Woodruff et al. 1997). A Taiwanese time-series study found a positive association between SIDS and reduced visibility during 1–9 days before death (Knöbel et al. 1995), but adjustment for confounders was limited. Although the U.S. cohort study found a significant association with PM_10_ (OR = 1.12; 95% CI, 1.07–1.17), the Czech case–control study did not find a significant association with TSP (OR = 0.91; 95% CI, 0.75–1.12) (Bobak and Leon 1999b).

#### Morbidity outcomes: respiratory morbidity.

The only study investigating respiratory morbidity in infants reported significant associations between exposure to PM_2.5_ and some (cough without infection and dry cough at night) but not other (wheeze, asthmoid or other types of bronchitis, respiratory infections, and sneezing, runny/stuffed nose) respiratory symptoms (Gehring et al. 2002).

## Discussion

### Main findings.

Our review suggests some evidence of an association between PM levels and different subgroups of infant mortality. There were differences in the magnitude and consistency of association by cause of death, with PNM due to respiratory causes and SIDS being more consistently associated with PM levels. However, it is problematic to compare cause-specific associations between studies because of variations in definitions and diagnostic criteria of causes of death. Differential findings for various mortality subgroups within some studies suggest a stronger association of particulate air pollution with some causes of infant death. The only study investigating respiratory morbidity in infants reported significant associations between exposure to PM_2.5_ and some but not other respiratory symptoms (Gehring et al. 2002).

### Methodologic issues.

We were unable to take publication, language, and reporting biases into account when identifying relevant publications, which may have overestimated the strength of any associations.

Summarizing the findings was complicated by the considerable differences in methodologies used. Many articles reported the results relating to a number of combinations of PM, outcome, and exposure period, resulting in multiple comparisons, which in turn increased the likelihood of positive findings.

More than half of the reviewed studies were ecologic or time series in design. Controlling for confounding factors in such studies is more difficult than in individual-based studies because of the extra potential sources of bias due to the aggregation of subjects into groups (Morgenstern and Thomas 1993). Even in semi-individual studies, few adjusted for key confounding factors at an individual level, because some used area-based level data and others did not adjust for confounders. Other important individual risk factors, such as smoking and environmental exposures from other air pollutants (e.g., sulfur dioxide, nitrogen dioxide), were rarely controlled for. Adequate control for confounders is essential to accurately estimate the magnitude of any association between low-level particulate air pollution exposure and infant health, and inadequate control may partly account for some inconsistency between studies.

Air pollution exposure was generally estimated by small numbers of monitors, which may not estimate individual exposures accurately for all infants; this could result in misclassification of exposure. The potential for bias is also affected by monitoring decisions (e.g., annual or daily means). The absence of information about indoor air pollution may underestimate individual exposure. These factors are likely to be nondifferential and therefore reduce the precision of effect estimates.

Studies exploring the health effects of PM may report inconsistent results because the definitions and measurement techniques are variable. The toxicity of equal-sized PM depends on its chemical composition, which, in turn, depends on the mixture of sources generating them and their dispersion (Mage 2002). For example, the PM_10_:TSP ratio ranges from 50 to 60% for U.S. sampling sites (Dockery and Pope 1994), whereas in the Czech Republic PM_10_ has been estimated to constitute about 80% of TSP (Bobak and Leon 1999a). The reviewed studies also varied in relation to average levels and ranges of PM, and copollutant exposures. Despite differences in air pollution sources and levels, the findings of an association between PM levels and postneonatal respiratory mortality are fairly consistent across studies and regions.

Another possible explanation for inconsistency of findings is differences between settings in the distribution of timing and cause of death within infant mortality. For instance, the definitions of respiratory causes of death and SIDS varied across studies (Bobak and Leon 1992, 1999b; Knöbel et al. 1995; Lipfert et al. 2000; Woodruff et al. 1997) and were not always fully reported (Lipfert et al. 2000). Accurate diagnosis of deaths due to SIDS depends on a postmortem investigation, and this was not available for all cases coded as SIDS in one study (Knöbel et al. 1995). For this reason, within-study comparisons, when different subgroups and causes of death were examined in the same study, may be more valid than between-study comparisons.

The magnitudes of association reported in the reviewed studies are low and could be accounted for by the factors considered above. However, their magnitude is of the same order as that found between PM and adult mortality, which is accepted as a true relationship (Committee on the Medical Effects of Air Pollution 1998; WHO 2002).

### Potential mechanisms.

Although the epidemiologic evidence linking increases in PM with excess mortality and morbidity in adults is now strong, the mechanisms for such a link are not yet well understood. To date, toxicologic studies have not identified unequivocally specific PM constituents or mechanisms to account for the epidemiologic observations. Infants and children are considered potentially susceptible populations in risk assessments, including risk from PM (U.S. EPA 1999), because of their immature immune system, potential impact on lung growth and development, and viral infections common in infants. However, few human and animal studies have compared immature and adult organisms with regard to their susceptibility to inhaled particles (Mauderly 2000). For adults, three potential mechanisms have been put forward for the PM effect: an inflammatory response that alters blood coagulation, an allergic immune response, and an alteration in cardiac autonomic function resulting in the reduction of heart rate variability (Donaldson et al. 2001; Granum and Lovik 2002; Liao et al. 2004; Pope 2000). All three potential mechanisms may be pertinent in infants, but the degree of their influence may vary at various stages of infant development. In particular, they may be more applicable to postneonatal death, because this is thought to be affected more by the infant’s external environment than is NM (Pharoah and Morris 1979). Neonatal deaths are more affected by conditions originating in the perinatal period, with immaturity-related conditions being the main cause of death. However, if there is a small adverse effect of particulate air pollution on fetal growth and duration of pregnancy, as discussed previously (Glinianaia et al. 2004), it may also indirectly contribute to neonatal deaths.

The mechanisms of SIDS, the most common cause of postneonatal death in developed countries, are not well understood, although a number of pathways have been proposed. One of the currently most compelling hypotheses for the occurrence of SIDS is an abnormality of brain development and maturation, with a tendency to central apnea and disturbed cardio-respiratory control mechanisms (Goldwater 2003; Harper 2000; Kahn et al. 2003; Kinney and Filiano 2001). Unsafe sleeping environment, exposure to environmental tobacco smoke (ETS), and lower socioeconomic status are critical risk factors for SIDS. It has been suggested that the association between post-natal exposure to tobacco smoke and SIDS is causal (Anderson and Cook 1997; McMartin et al. 2002). The potential mechanisms of action proposed for ETS (abnormal pulmonary development, reduced pulmonary function, abnormalities in cardiorespiratory control system, promotion of respiratory infections) (Chan-Yeung and Dimich-Ward 2003; Goldwater 2003; Hofhuis et al. 2003; Strachan and Cook 1997) might be similar to those for particulate air pollution, because ETS is known to contain a substantial proportion of PM.

### Implications.

Current epidemiologic evidence suggests a link between ambient PM exposure and some subgroups of infant mortality, even at relatively low PM levels reported in the reviewed studies, which are comparable with current levels experienced in developed countries. More research is needed to further clarify whether there is a real effect of particulate air pollution on infant health and to quantify this effect. Future studies should explore overall and cause-specific infant mortality, using robust study designs with individual level information on key confounding variables. Exposure assessment should include details of level, size, and composition of PM and co-pollution exposure. The use of physiologic measurements (e.g., lung function in older children) and biomarkers of exposure or effect (e.g., methemoglobin as a biomarker of carbon monoxide poisoning, cotinine as a marker of exposure to ETS, placental DNA adduct levels as biomarkers of effect of polycyclic aromatic hydrocarbons) could promote understanding of causal effects of air pollution on infant and children’s health. If a causal association between exposure to PM and infant death exists, widespread exposure to particulate air pollution may be an important determinant of infant mortality at a population level.

## Figures and Tables

**Table 1:** Studies investigating the association between PM and infant mortality.

Reference, country, data collection period	Study design, sample size, exposure period(s) considered	Estimate (95% CI) by exposure and type of mortality	Adjustment for confounding factors
**Studies exploring multiple outcomes**
Bobak and Leon 1999b	Matched case–control	Per 10-μg/m^3^ increase in TSP:	Socioeconomic factors, maternal age and parity, gestational age, BW, birth length
Czech Republic 1989–1991	2,006 cases and 7,952 controls with data on TSP	Total infant: AOR = 1.03 (0.99–1.06)	
		Infant respiratory: AOR = 1.12 (1.01–1.28)^a^	
		Infant nonrespiratory: AOR = 1.01 (0.98–1.05)	
		Total neonatal: AOR = 1.00 (0.96–1.06)	Also adjusted for other pollutants examined (SO_2_ and NO*_x_*), but the results are not given
		Neonatal respiratory: AOR = 0.93 (0.67–1.32)^a^	
		Neonatal nonrespiratory: AOR = 1.00 (0.96–1.06)	
	Mean over period between birth and death	Total postneonatal: AOR = 1.04 (0.99–1.10)	
		Postneonatal respiratory: AOR = 1.14 (1.02–1.32)^a^	
		Postneonatal nonrespiratory: AOR = 1.02 (0.96–1.08)	
		SIDS: AOR = 0.91 (0.75–1.12)	
Lipfert et al. 2000	Cross-sectional	Per 10-μg/m^3^ increase in PM_10_:	Socioeconomic factors, mother’s smoking, month of birth
USA 1990	1,443,768 births and 13,041 infant deaths; 2,354 infant deaths due to respiratory causes^b^	Total infant: AOR = 1.12 (1.09–1.15)	
		Infant respiratory: AOR = 1.18 (1.11–1.26)^b^	Not adjusted for other pollutants examined (SO_2_, CO, SO_4_^2–^)
		AOR = 1.14 (0.96–1.35)^a^	
		Total neonatal: AOR = 1.13 (1.09–1.18)	
	341 infant deaths due to respiratory causes^a^	Neonatal respiratory: AOR = 1.17 (1.09–1.26)^b^	
	8,362 neonatal deaths; 4,679 postneonatal deaths 1,918 SIDS deaths	Total postneonatal: AOR = 1.10 (1.04–1.15)	
		Postneonatal respiratory: AOR = 1.21 (1.05–1.39)^b^	
		SIDS: AOR = 1.15 (1.07–1.24)	
	Annual mean		
Bobak and Leon 1992	Ecologic	> 84.7 (top quintile) vs. < 53.6 μg/m^3^ TSP (bottom quintile):	Socioeconomic factors
Czech Republic 1986–1988	~22,370 live births 2,699 infant deaths	Total neonatal: AOR = 1.18 (1.00–1.39), test for trend *p* = 0.071	Also adjusted for other pollutants examined (SO_2_, NO*_x_*), but the results are not given
	Annual mean	Total postneonatal: AOR = 1.53 (1.20–1.97), test for trend *p* < 0.001	
		Postneonatal respiratory: AOR = 3.16 (1.52–6.55),^a^ test for trend *p* = 0.001	
Woodruff et al. 1997	Cohort	Per 10-μg/m^3^ increase in PM_10_:	Socioeconomic factors, mother’s smoking, month of birth
USA 1989–1991	All infants: 3,788,079	Total postneonatal: AOR = 1.04 (1.02–1.07)	
		Postneonatal respiratory mortality in normal-BW infants: AOR = 1.20 (1.06–1.36)^a^	
	First 2 months of life		
		Postneonatal respiratory in LBW infants:	
		AOR = 1.05 (0.91–1.22)^a^	
		Postneonatal nonrespiratory:	
		AOR = 1.00 (0.99–1.00)	
		SIDS: AOR = 1.12 (1.07–1.17)	
Ha et al. 2003	Time series	Per 10-μg/m^3^ increase in PM_10_:	Seasonality, temperature, relative humidity, day of week
South Korea 1995–1999	1,045 postneonatal deaths	Total postneonatal: ARR = 1.03 (1.02–1.04)	
	Daily mean on event day	Postneonatal respiratory: ARR = 1.18 (1.14–1.21)	
Chay and Greenstone 1999	Ecologic	Total infant mortality: a 10-μg/m^3^ increase in TSP associated with 3.5 more deaths per 10,000 live births (SE = 1.9)	Maternal, infant, and socioeconomic factors, and weather data (measured at county level)
USA 1980–1982	101,730 infant deaths of > 8.5 million births		
	70,649 neonatal deaths	Total neonatal mortality: a 10-μg/m^3^ increase in TSP associated with 3.4 more deaths per 10,000 live births (SE = 1.7)	
	Changes in annual mean		
	TSP induced by recession		
Chay and Greenstone 2003	Ecologic	Total infant mortality: a 10-μg/m^3^ increase in TSP associated with 13 more deaths per 10,000 live births (SE = 5.6)	Maternal, infant, and socioeconomic factors (measured at county level)
USA 1971–1972	> 4 million births in 489 U.S. counties		
		Total neonatal mortality: a 10-μg/m^3^ increase in TSP associated with 6.6 more deaths per 10,000 live births (SE = 4.4)	
	Change in annual mean TSP induced by Clean Air Act		
Penna and Duchiade 1991	Ecologic	Total infant mortality: no significant association with TSP level	Socioeconomic factors
Brazil 1980	Sample size not given		
		Infant mortality due to pneumonia: a 10-μg/m^3^ increase in average PM level associated with 2.2 more deaths per 10,000 live births, *p* = 0.014	
	Annual geometric mean and the year’s daily maximum		
		No significant association with annual maximum TSP	
Lave and Seskin 1972	Ecologic	Total infant mortality: a 10-μg/m^3^ increase in minimum TSP level associated with 3.4 more deaths per 10,000 live births, *p* < 0.05	Socioeconomic factors, weather
USA 1960	Minimum biweekly level		
	Annual mean	Total neonatal mortality: a 10-μg/m^3^ increase in TSP associated with 0.66 more deaths per 10,000 live births, *p* > 0.05	
	Exact sample size not given (based on 117 SMSAs)		
**Studies exploring one outcome only**
**Total infant mortality**
Loomis et al. 1999	Time series	Per 10-μg/m^3^ increase in PM_2.5_: lag 3–5 days; ARR = 1.069 (1.025–1.113)	Mean temperature of the 3 days before death. Also adjusted for other pollutants examined (O_3_, NO_2_), but the results are not given
Mexico 1993–1995	2,798 infant deaths 0–6 days before death		
Hunt and Cross 1975	Cross-sectional	Higher risk of IM in one of four 3-month periods (with reported air pollution episodes), *p* < 0.001	No adjustment for any confounders
USA 1970	66 infant deaths in 3,739 live births		
		Proportion of IM from respiratory causes was higher in same 3-month period (*p* = 0.02)	Primarily descriptive statistics
	Exposure period not specified		
**Total neonatal mortality**
Joyce et al. 1989	Ecologic	Per 10-μg/m^3^ increase in TSP: AOR = 1.04 (*p* > 0.05)	Low BW
USA 1976–1978	Sample size not given 4-year mean (1975–1978)		
Shinkura et al. 1999	Time series	Per 10-μg/m^3^ increase in TSP: RR = 1.01 (0.99–1.04)	Season, calendar year, sex, but data not reported
Japan 1978–1988	98 neonatal deaths in ~29,790 live births		
	Mean of birth month		
**SIDS**
Knöbel et al. 1995	Time series	For visibility 1–3 km vs. 22–37 km:	Weather, season, population size, level of urbanization, daily incidence of respiratory tract infections
Taiwan 1981–1991	3,816,000 live births	ARR on day of death = 3.8 (2.8–5.1), ARR during 9 days before death = 5.1 (3.2–8.1)	
	3,005 deaths (estimated based on the crude rate)		
	Day of death, 1–9 days before death		

Abbreviations: AOR, adjusted odds ratio; ARR, adjusted rate ratio; BW, birth weight; IM, infant mortality; LBW, low birth weight, < 2,500 g; RR, relative risk; SIDS, sudden infant death syndrome; SMSAs, standard metropolitan statistical areas. Values in parentheses after AOR or ARR are 95% CI. Where possible, study results were re-expressed as the estimated effect of increasing air pollution levels by 10 μg/m^3^ (TSP, PM_10_, PM_2.5_). PNM due to respiratory causes identified by the *International Classification of Diseases*, 9th Revision (ICD-9; World Health Organization 1977).

a ICD-9 codes 460–519 (Bobak and Leon 1999b; Lipfert et al. 2000; Woodruff et al. 1997).

b ICD-9 codes 460–519 and 769, 770 (Lipfert et al. 2000). SIDS deaths were defined as those with an ICD-9 cause code of 798.0 (Bobak and Leon 1999b; Woodruff et al. 1997); in one study the SIDS cases also included deaths from suffocation (Knöbel et al. 1995).
